# Endogenous opioids in the olfactory tubercle and their roles in olfaction and quality of life

**DOI:** 10.3389/fncir.2024.1408189

**Published:** 2024-05-30

**Authors:** Koshi Murata, Ayako Maegawa, Yoshimasa Imoto, Shigeharu Fujieda, Yugo Fukazawa

**Affiliations:** ^1^Division of Brain Structure and Function, Faculty of Medical Sciences, University of Fukui, Fukui, Japan; ^2^Life Science Innovation Center, University of Fukui, Fukui, Japan; ^3^Department of Otorhinolaryngology-Head and Neck Surgery, Faculty of Medical Sciences, University of Fukui, Fukui, Japan

**Keywords:** olfactory tubercle, opioid, quality of life, prodynorphin, preproenkephalin, dopamine, brain reward system

## Abstract

Olfactory dysfunctions decrease daily quality of life (QOL) in part by reducing the pleasure of eating. Olfaction plays an essential role in flavor sensation and palatability. The decreased QOL due to olfactory dysfunction is speculated to result from abnormal neural activities in the olfactory and limbic areas of the brain, as well as peripheral odorant receptor dysfunctions. However, the specific underlying neurobiological mechanisms remain unclear. As the olfactory tubercle (OT) is one of the brain’s regions with high expression of endogenous opioids, we hypothesize that the mechanism underlying the decrease in QOL due to olfactory dysfunction involves the reduction of neural activity in the OT and subsequent endogenous opioid release in specialized subregions. In this review, we provide an overview and recent updates on the OT, the endogenous opioid system, and the pleasure systems in the brain and then discuss our hypothesis. To facilitate the effective treatment of olfactory dysfunctions and decreased QOL, elucidation of the neurobiological mechanisms underlying the pleasure of eating through flavor sensation is crucial.

## Introduction

The emergence of coronavirus disease, which is often accompanied by olfactory dysfunctions or degeneration, has led to the need for re-evaluation of the impact of olfaction in quality of life (QOL) (Coelho et al., 2021). Olfactory dysfunctions can arise from various causes, such as viral infections, allergic rhinitis, and trauma (Hummel et al., 2017; Miwa et al., 2019). Olfactory dysfunction decreases daily QOL, with reduced food enjoyment as a significant factor (Croy et al., 2014). Olfaction is a key component of flavor sensation; when food is masticated and swallowed food, odorants are detected via the olfactory epithelium through the retronasal pathway, wherein olfactory sensory neurons send food odorant signals the brain (Lawless, 1991; Shepherd, 2011; Shiotani et al., 2024). Why does the inability to sense flavor affect the pleasure of eating? In other words, which neurobiological mechanisms connect flavor sensations and pleasure of eating? The key factors of decreased QOL owing to olfactory dysfunction have been considered as changes in the activity of olfactory and limbic neural circuits (Rochet et al., 2018). Here, we hypothesized that the endogenous expression of opioids in the olfactory tubercle (OT) is involved in the neurobiological mechanisms underlying pleasure sensation through olfaction while eating (Maegawa et al., 2022). In this review, we provide an overview of the recent updates on the structure and function of the OT, briefly summarize the endogenous opioid system in the mammalian brain, and review the gene expression of prodynorphin and preproenkephalin in the OT. We, then, explore the pleasure systems in the brain, emphasizing the involvement of opioids in hedonic sensations. Finally, we discuss the hypothesis that olfactory dysfunctions decrease daily QOL by suppressing opioid release from the anteromedial subregion of the OT while eating.

## Structure and functions of the OT

The OT is a part of the ventral striatum and is anatomically continuous with the nucleus accumbens (NAc), a chief target of the mesolimbic dopaminergic pathway (Heimer, 1978; Heimer et al., 1987; Ikemoto, 2007). The term “olfactory tubercle” refers to the region on the basal surface of the frontal lobe between the olfactory tract and nucleus of the diagonal band in humans (Crosby and Humphrey, 1941; Allison, 1954; Sakamoto et al., 1999). The OT receives dense synaptic inputs from the central olfactory areas, such as the olfactory bulb, anterior olfactory nucleus, tenia tecta, and piriform cortex, which may transmit olfactory information to the OT (Haberly and Price, 1977, 1978a,b; Berendse et al., 1992). In addition, the OT receives synaptic inputs from the prefrontal cortex, amygdala, and hypothalamus as well as dopamine input from the ventral tegmental area (VTA) (Ikemoto, 2007; Zhang et al., 2017a,b). Despite its olfactory label, the OT exhibits multimodal sensory responsiveness that is not limited to olfaction (Wesson and Wilson, 2011).

The principal neurons of the OT comprise three major subtypes: the medium spiny neurons (MSNs), dwarf cells, and granule cells, all of which are GABAergic (Millhouse and Heimer, 1984; Xiong and Wesson, 2016). MSNs are distributed throughout the OT, forming a dense cell layer (also referred to as layer II). OT MSNs project their axons primarily onto the ventral pallidum (Heimer et al., 1987; Walaas and Ouimet, 1989; Zhou et al., 2003; Lee et al., 2023). Dwarf cells are clustered in the lateral part of the OT, forming the cap region that is interspersed throughout the anteroposterior axis (Hosoya and Hirata, 1974). Granule cells are clustered from the anteromedial surface to the deep layers of the central OT, forming a continuous structure of the Islands of Calleja (ICj) (Fallon et al., 1978; de Vente et al., 2001). The axonal targets of the granule cells are MSNs in the OT, which project their axons to the VTA, suggesting the role of granule cells in the disinhibition of dopamine release from the VTA (Zhang et al., 2023). Dopamine receptor subtypes are expressed differently by these principal neurons: MSNs express D1 or D2 dopamine receptors, dwarf cells express D1 receptors, and granule cells express D3 receptors (Le Moine and Bloch, 1995; Yung et al., 1995; Murata et al., 2015; Zhang et al., 2021).

The anatomically distinct domains of the OT play different roles in physiological and behavioral responses (Yamaguchi, 2017; Murata, 2020). In previous studies, reward motivation functions of the OT were implicated by intra-cranial self-administration of addictive drugs (Ikemoto, 2010; Berridge and Kringelbach, 2015). The anteromedial domain of the OT in particular support self-administration in rats (Ikemoto, 2003; Ikemoto et al., 2005; Shin et al., 2008), and its involvement in odor-guided appetitive behaviors also has been revealed. Specifically, behavioral attraction to an odor cue associated with sugar-reward induces c-fos activation in the anteromedial OT (Murata et al., 2015). Optogenetic and DREADD manipulations of dopamine inputs from the VTA to medial OT have revealed this pathway to mediate the acquisition and execution of odor preference (Zhang et al., 2017a). The cytochemical architecture of the anteromedial OT develops postnatally, and the response of c-fos expression to eating matures during the late weaning period (Murofushi et al., 2018). Conversely, the lateral OT is involved in odor-guided aversive behaviors. Aversive behavioral response to an odor cue associated with electrical foot shock induces c-fos activation of MSNs and dwarf cells in the lateral OT (Murata et al., 2015). Projection neurons responsive to predator odors and rotten food odors in the olfactory bulb project their axons to the cap region in the lateral OT (Igarashi et al., 2012). Additionally, optogenetic activation of predator odor-responsive glomerulus in the olfactory bulb induces aversive behavior and Egr1 activation in the cap region (Saito et al., 2017). Associative learning of conditioned odor stimuli with distinct sugar rewards, or with electrical foot shocks, shapes the responsiveness of the anteromedial and lateral OT domains, respectively (Sha et al., 2023). The altered response of the OT domains is accompanied by increased axonal bouton size and higher expression of the excitatory synaptic structure (VGluT1) in the axon terminals from the olfactory bulb and anterior piriform cortex to the OT (Sha et al., 2023). In addition to regional differences, the subtypes of OT neurons play different physiological and behavioral roles. Optogenetic activation of D1-expressing MSNs in the anteromedial OT elicits place preference, whereas activation of D2-expressing MSNs in the same site elicits place aversion (Murata et al., 2019). Further, D3-expressing granule cells in the ICj are involved in grooming behavior and suppression of depression-like behaviors (Zhang et al., 2021, 2023).

Mesolimbic dopamine circuits play crucial roles in incentive motivation, and also reinforcement learning and valence coding (Schultz, 1998, 2016), and a series of studies have revealed the role of the OT in this context (Gadziola et al., 2015; Millman and Murthy, 2020; Oettl et al., 2020; Winkelmeier et al., 2022). Distinct roles for the subtypes of D1- and D2-expressing neurons of the OT in reinforcement learning have also been suggested but remain controversial. Gadziola et al. (2020) demonstrated that D1-expressing neurons flexibly represent rewarded odors during reversal learning, and activation of D1-expressing neurons promotes engagement in a reward-motivated task. Martiros et al. (2022) validated the role of D1-expressing neurons by demonstrating that they robustly and bidirectionally represent odor valence, responding similarly to odors that predicted similar outcomes, regardless of odor identity. Martiros et al. (2022) also showed that D2-expressing neurons were conversely more selective for odor identity than valence. However, Lee et al. (2023) proposed that although D1-expressing neurons show larger response magnitudes to rewarded odors than other odors, this is better interpreted as identity encoding with enhanced contrast rather than value encoding. As the OT has a laterality of anatomical and functional domains, future studies should be conducted for both cell types (D1 vs. D2) and anatomical domains (medial vs. lateral) to clarify the roles of OT neurons in valence coding and odor identity. In summary, the OT is part of the ventral striatum and plays a crucial role in motivating animals to acquire and execute adaptive behaviors through valence coding and reinforcement learning.

## Endogenous opioid systems in the mammalian brain and expression of dynorphins and enkephalins in the OT

The opioid system is a highly diverse peptide-neurotransmitter system that provides pain relief and euphoric effects (Emery and Akil, 2020). Endogenous opioids are composed of three main neuropeptide families: the β-endorphins derived from the pro-opiomelanocortin gene (*Pomc*), the enkephalins derived from preproenkephalin (*Penk*) and prodynorphin (*Pdyn*) genes, and the dynorphins derived from the *Pdyn* gene. Each of these families contains multiple peptides with diverse binding characteristics (Przewlocki, 2022). The system includes three opioid receptors: the mu, delta, and kappa. The opioid peptides have been portrayed to bind primarily to particular receptors (β-endorphins: mu, enkephalins: delta, and dynorphins: kappa); however, the lack of one-to-one correspondence between signal peptides and its receptors should be emphasized. Members of all three peptide families are capable of activating the three receptors to varying degrees, particularly the dynorphins (Emery and Akil, 2020). Therefore, all opioid receptor types within a brain region may simultaneously be activated by different or by even the same peptide. A fourth receptor-peptide pair, nociceptin and its receptor, are also part of the opioid system and play roles in pain and motivation (Di Cesare Mannelli et al., 2015; Kiguchi et al., 2016; Parker et al., 2019). This pair was more recently discovered than the others and is not reviewed here (Mollereau et al., 1994; Meunier et al., 1995).

**Figure 1:**
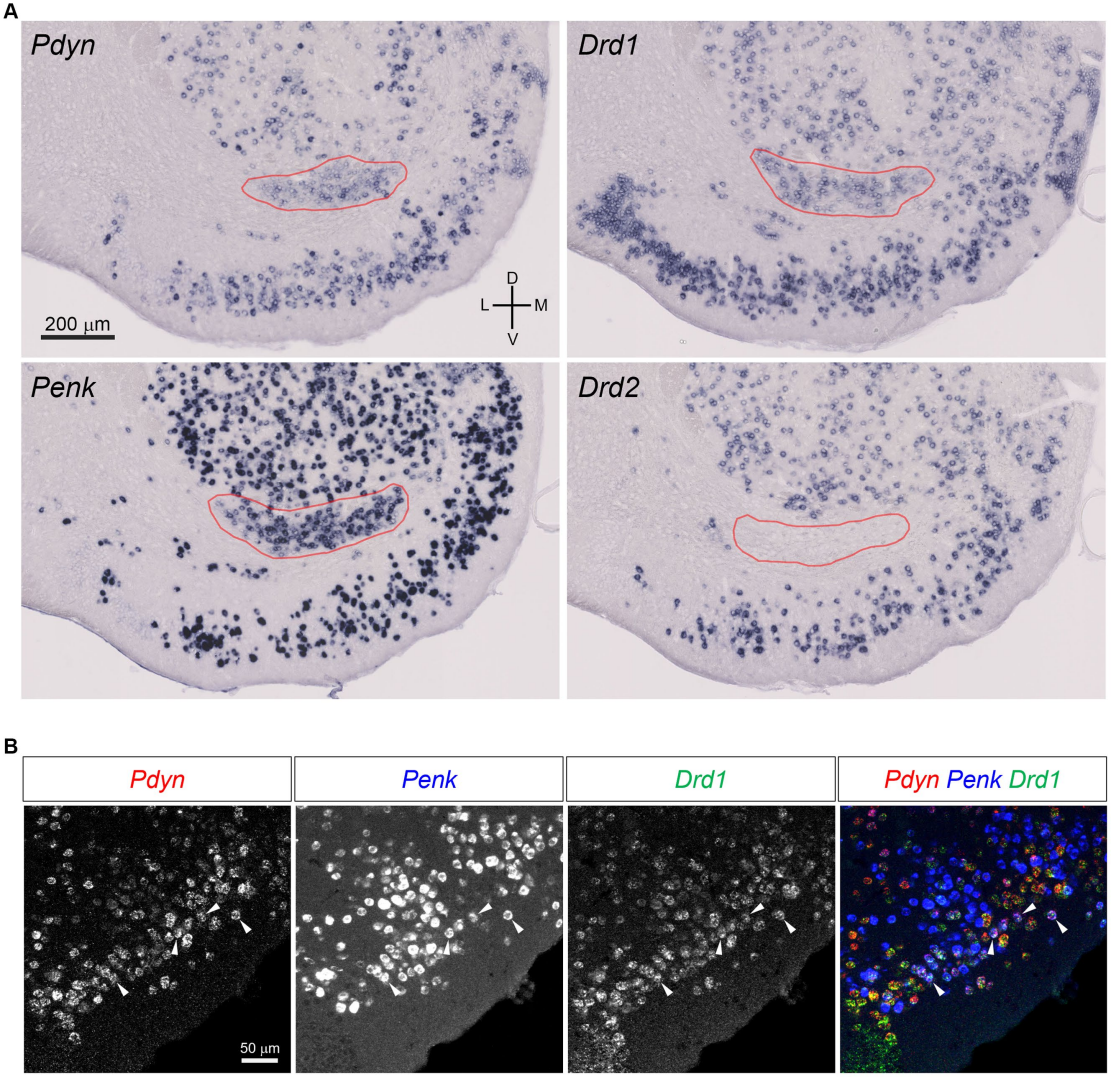
*Pdyn*-*Penk* co-expressing D1 neurons in the mouse OT. **(A)** Single probe *in situ* hybridization for *Pdyn*, *Penk*, *Drd1*, and *Drd2*. The pictures show coronal sections of the anterior OT and NAc (approximately at Bregma +1.94 mm). Regions delineated by red lines are a cluster of *Pdyn-Penk-Drd1* co-expressing cells. *Drd2* signals were not observed in the cluster. Adjacent sections from one mouse were used for the four images. **(B)** Triple fluorescence *in situ* hybridization for *Pdyn*, *Penk*, and *Drd1* in the anteromedial OT. White arrowheads indicate the colocalization of *Pdyn-Penk-Drd1* mRNAs. D, dorsal; V, ventral; M, medial; L, lateral. Figures are modified from Maegawa et al. (2022).

*Pomc* expression in the brain is limited to the arcuate nucleus of the hypothalamus, nucleus of the solitary tract, and pituitary gland (Bronstein et al., 1992). The regions that express *Pdyn and Penk* are widely distributed in the brain (Harlan et al., 1987; Merchenthaler et al., 1997), and their details have not been included in this review. Here, we focused on the striatum, one of the brain’s regions with high *Pdyn* and *Penk* expression (Figure 1A). Neurons in the dorsal striatum and NAc generally express either *Pdyn* or *Penk* (Furuta et al., 2002); *Pdyn* is expressed by D1 neurons and *Penk* by D2 neurons (Curran and Watson, 1995). The ventral striatum has a cell cluster between the NAc and OT, where D1 neurons co-express *Pdyn* and *Penk*, and no D2 neurons are observed (Curran and Watson, 1995). In a recent report, we evaluated the cellular profiles of *Pdyn*- and *Penk*-expressing neurons in mouse OT (Maegawa et al., 2022). These results are consistent with those of the dorsal striatum and NAc, in that D1 neurons express *Pdyn* and D2 neurons express *Penk*. In addition, we found that the OT had D1 neurons co-expressing *Pdyn* and *Penk* in the dense cell layer and a higher frequency of co-expressing D1 neurons in the anteromedial OT than in the anterolateral OT (Figure 1B).

The three types of opioid receptors are widely distributed in the brain and show a high degree of overlap in their regional expression patterns, particularly in regions involved in pain, affect, and rewards (Mansour et al., 1988; Mansour and Watson, 1993). Describing the details of the opioid receptor distribution is beyond the scope of this review. We focused on brain regions with mu, delta, and kappa receptor expression that are positionally proximate to the OT and have axonal projections from the OT, as demonstrated by *in situ* hybridization of mRNA and peptide binding assays (Mansour et al., 1987, 1994). Mu receptor expression is localized in the caudate putamen, NAc, lateral and medial septa, diagonal band of Broca, bed nucleus of the stria terminalis, thalamus, habenula, interpeduncular nucleus, and substantia nigra. Delta receptor expression is found in the caudate putamen, NAc, OT, ventromedial hypothalamus, and amygdala. Kappa receptor expression is localized in the claustrum, endopiriform nucleus, caudate putamen, NAc, OT, medial preoptic area, bed nucleus of the stria terminalis, amygdala, hypothalamus, periventricular thalamus, substantia nigra, and ventral tegmental area.

## Implications of the OT opioids on the pleasure of eating and QOL

Opioids are neurochemical molecules related to feelings of pleasure (Barbano and Cador, 2007). Dopamine is often misunderstood as a pleasure mechanism in the brain (Berridge and Dayan, 2021). Our understanding of the brain’s pleasure system can be traced back to the discovery of intracranial self-stimulation in rats by Olds and Milner (1954). Olds (1956) had originally interpreted that the rats engaged in intense lever pressing to self-stimulate because the electrical stimulation of the brain was pleasurable. Subsequent studies have since demonstrated that spontaneous approaching and reward-seeking behaviors such as lever pressing for a reward involve the dopamine system (Wise et al., 1978). Therefore, dopamine was initially considered to be a pleasure substance (Wise, 1980); however, later studies suggested that appetitive behavior due to dopamine activation or hypothalamic electrical stimulation should be interpreted as reflecting motivated “wanting” but not necessarily pleasurable hedonic impact or “liking” (Berridge and Robinson, 2016). To objectively measure an animal’s hedonic impact, an affective taste reactivity test is widely used whereby a sweet sucrose solution is passively infused into the animal’s oral cavity, and its facial and bodily movements are observed. Typical responses to sweet sucrose infusion are intaking behaviors such as rhythmic tongue protrusions, and used as objective measurement of hedonic “liking” reactions (Berridge, 2000; Steiner et al., 2001). Ablation of the dopamine neurons in the VTA does not reduce the hedonic “liking” reaction to the sucrose oral infusion, despite abolishing the animal’s voluntary food and water intake (Berridge et al., 1989). Furthermore, electrical stimulation of the lateral hypothalamus that activates dopamine neurons does not increase the hedonic “liking” reaction, despite dramatically increasing food intake (Berridge and Valenstein, 1991). At present, dopamine is not considered a pleasure-inducing substance but rather is related to motivation, craving, and prediction error for learning adaptive behavior (Wise, 2008; Kringelbach, 2010; Berridge and Kringelbach, 2015; Schultz, 2016).

In contrast, opioids are neurotransmitters, and their agonists enhance and antagonists block hedonic “liking” reactions associated with intake behaviors such as rhythmic tongue protrusions in response to sucrose sweetness. Blocking the opioid system by systemic naltrexone injection inhibits the hedonic “liking” reactions (Parker et al., 1992), and systemic morphine injection increases the hedonic “liking” reactions (Doyle et al., 1993). In addition, the microinjection of opioids into the rostrodorsal NAc medial shell, caudal VP, caudal insular cortex, and rostromedial orbitofrontal cortex enhances the hedonic reactions of “liking” to sucrose taste (Peciña and Berridge, 2005; Smith and Berridge, 2007; Castro and Berridge, 2014, 2017); these local subregions are known as hedonic hotspots (Morales and Berridge, 2020). The dynorphins-kappa receptor system generally known to induce unpleasantness when systemically administrated, but can oppositely enhance the “liking” reactions when a kappa agonist is locally injected into the NAc hedonic hotspot, similar to mu and delta agonist microinjections (Castro and Berridge, 2014). The OT is neuroanatomically and neurochemically similar to the NAc in that both have GABAergic projections to the VP and express *Pdyn* and *Penk*. Studies have demonstrated that OT plays a crucial role in reward seeking-motivated behavior, a hallmark of the dopamine-related brain reward system (Ikemoto, 2003; Murata et al., 2015; Zhang et al., 2017a). Having a hedonic hotspot is another feature of some brain reward structures (Morales and Berridge, 2020), which raises the possibility that the OT may have its own hedonic hotspot where opioid stimulation might enhance “liking” reaction to sucrose sweetness.

**Figure 2:**
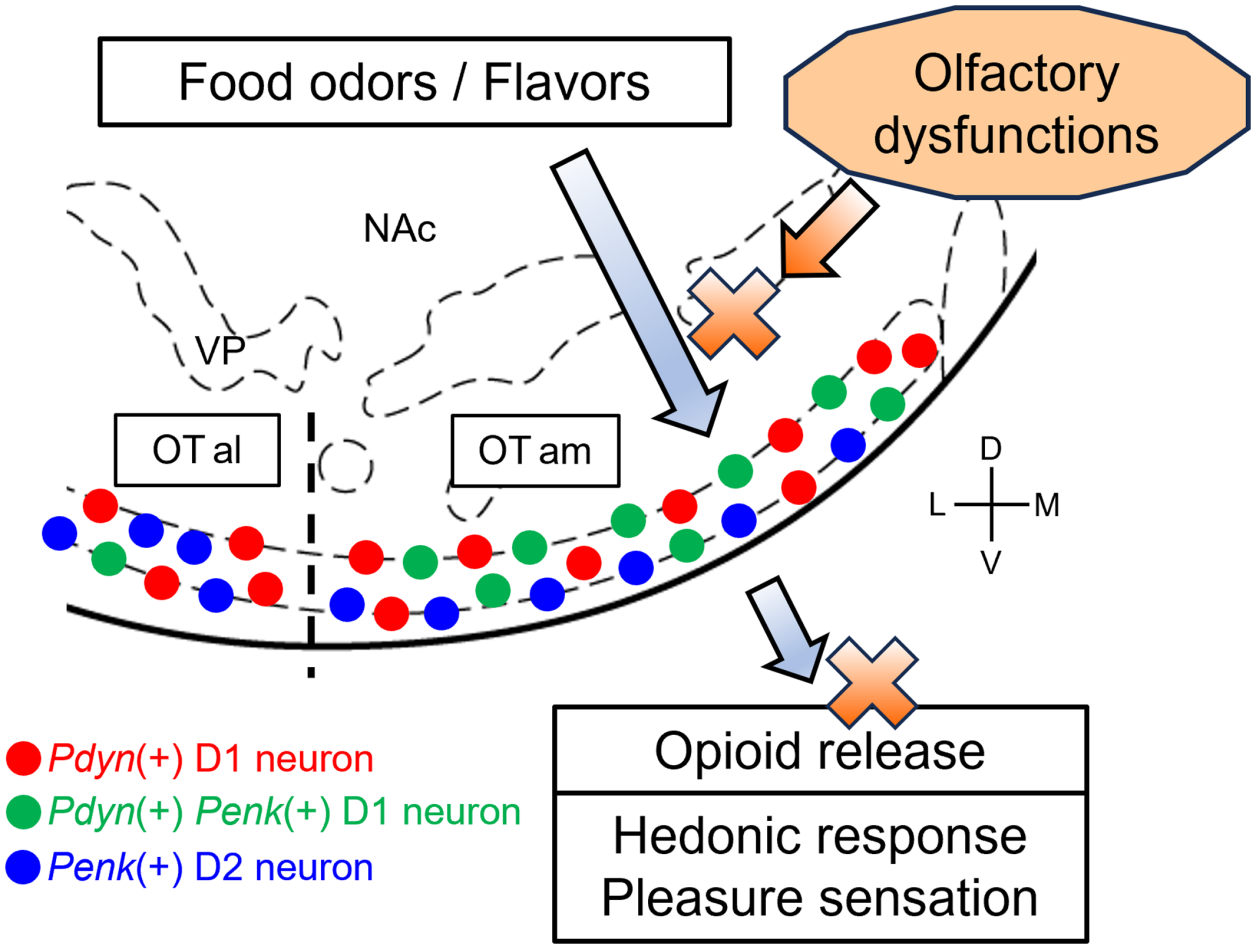
A hypothetical model of OT opioid-mediated QOL and its impairment due olfactory dysfunctions. In a healthy condition, food-related olfactory inputs, including food odorants via the retronasal pathway, are conveyed to the anteromedial OT, where *Pdyn*-expressing and *Pdyn-Penk*-coexpressing D1 neurons are activated. Subsequently, opioid peptides are released from D1 neurons in the anteromedial OT, resulting in hedonic pleasure sensation. In some cases of olfactory dysfunctions, the retronasal airflow is blocked, inhibiting the transmission of the olfactory inputs to the anteromedial OT. This, in turn, impairs neural activation and opioid release of the anteromedial OT, which reduces the sensation of pleasure. Red circles, *Pdyn*-expressing D1 neurons; green circles, *Pdyn-Penk*-coexpressing D1 neurons; blue circles, *Penk*-expressing neurons D2. OTam, anteromedial olfactory tubercle; OTal, anterolateral olfactory tubercle; NAc, nucleus accumbens; VP, ventral pallidum; D, dorsal; V, ventral; M, medial; L, lateral. Stereotax atlas from Franklin and Paxinos (2008).

Thus, we propose the following hypothesis: odorants in food are detected by olfactory neurons in the olfactory epithelium through the retronasal pathway and signaled to the brain, eliciting flavor sensations. Simultaneously, the signals of these odorants are transmitted to the anteromedial OT, inducing the release of dynorphins and enkephalins and resulting in a sensation of pleasure. In olfactory dysfunctions, retronasal airflow is blocked, inhibiting the first step of this cascade. It then suppresses both activation and opioid release in the anteromedial OT, which reduces the pleasure of eating (Figure 2). The anteromedial OT contains *Pdyn*-*Penk*-coexpressing D1 neurons, which may effectively induce pleasure, as well as appetitive motivation, in response to food odorants and flavors. Whether food odorants are detected by the anteromedial OT via the retronasal pathway remains unclear; therefore, we speculate this based on the analogy that food-related odors and actual food intake activate the anteromedial OT (Murata et al., 2015; Murofushi et al., 2018; Sha et al., 2023). Al-Hasani et al. (2018) demonstrated *in vivo* detection of optically evoked endogenous opioid peptide release by neurons in the NAc, supporting the idea that neural activation of OT neurons leads to the release of dynorphins and enkephalins. Midroit et al. (2021) demonstrated a correlation between the pleasantness of odors and the activity of the OT in humans. Future studies should investigate the relationship between the pleasantness of odors, neural activity of the OT, and opioid release.

## Discussion

We hypothesize that endogenous opioids in the OT, especially in the anteromedial domain, may contribute to neural processing of the pleasure of eating through flavor sensations. Olfactory dysfunction may lead to the disrupted processing within the OT, potentially reducing the daily QOL. The following crucial questions should be addressed in future to test this hypothesis: is the anteromedial OT able to enhance hedonic “liking” reactions to oral sucrose infusion? Does the flavor sensation of food stimulate the anteromedial OT and elicit opioid local release? Which neural circuits are targeted by opioids released from the anteromedial OT? What are the specific roles of *Pdyn*-*Penk*-coexpressing D1 neurons in the anteromedial OT compared with other types of OT neurons?

Although this review has focused on the endogenous opioids, we did not aim to de-emphasize the involvement of other neurotransmitters, including dopamine, in decreased QOL due to olfactory dysfunctions. As mentioned earlier, dopamine is a key neurotransmitter underlying incentive motivation for rewards (Wise, 2004). A lack of motivation is another symptom that accompanies olfactory dysfunctions, significantly affecting daily QOL (Keller and Malaspina, 2013; Marin et al., 2023). Olfactory dysfunctions can go beyond decreased QOL: they are bi-directionally associated with psychiatric disorders, including depression (Kohli et al., 2016; Taquet et al., 2021; Hasegawa et al., 2022). Ablation or inhibition of D3-expressing granule cells in the OT leads to depression-like behaviors and negatively impacts dopamine release from the VTA (Zhang et al., 2023). Conversely, olfactory enrichment has been shown to be a beneficial treatment strategy for depression (Leon and Woo, 2022). OT neural activity enhancement could be a treatment approach for depression through restoration of appropriate dopamine activity. Given that the OT is a hub region within mesolimbic dopamine neural circuits (Ikemoto, 2007), it likely plays important roles in both opioid-regulated pleasure sensation and dopamine-regulated motivation (Morales and Berridge, 2020). Further clarification of the neurobiology of the OT under olfactory dysfunctions is essential for developing more effective treatments for decreased QOL.

## Author contributions

KM: Writing – review & editing, Writing – original draft, Supervision, Conceptualization. AM: Writing – review & editing, Writing – original draft, Conceptualization. YI: Writing – review & editing. SF: Writing – review & editing. YF: Writing – review & editing.
